# Abstracts and Gleanings

**Published:** 1886-03-20

**Authors:** 


### ABSTRACTS AND GLEANINGS.

**Hyoscine Hydrobromate.**—(Therapeutic Gazette) Dr. Henry M. Wetherill has been employing hyoscine hydrobromate as an hypnotic for the past six months in the Pennsylvania Hospital for the Insane, and he publishes the results of his experience in the Medical Times, December 26, 1885. He used a formula containing 1 grain of the hydrobromate of hyoscine; 9 fluidrachms of distilled water; and a drachm of alcohol, every 10 minims of this solution containing 1-60 of a grain of hyoscine. As a hypnotic, the usual range of dose is from 1 120 to 1-90 of a grain given at bedtime, preferably by the mouth; very frequently a less dose than gr. 1-120 will be sufficient; gr. 1-200 has often acted better in insomnia than has a larger quantity. It is very seldom necessary to repeat a dose; and another very decided advantage possessed by hyoscine over hyoscamine is that small doses can be continued for a long time without increase; whereas the patient soon tolerates small and then moderate quantities of hyoscyamine, and finally resists very large doses of it. Dr. Wetherill has given hyoscine a thorough trial in the insomnia occuring in the course of acute delirious mania, and with marked success, having succeeded when all the usual modes of treatment had proved inadequate, in securing for the patient from six to ten hours of quiet sleep nightly for the past nine weeks, with but four or five exceptions, giving but one dose in every twenty-four hours, at bed time, the amount ranging from gr. 1-120 to 1-70. The insomnia in these cases is one of their chief elements of danger. If it is possible to give such a case a fair amount of sleep and of nourishment in a concentrated form, the probability of a favorable issue might be entertained. In the insomnia of agitated melancholia, of the morphine habit, of alcoholism, of acute mania, of neurasthenia, of chronic mental disorder, with habitual wakefulness and motor activity, and in those confirmed cases of insomnia from unascertained cause which usually prove so obnoxious to treatment, hyoscine has been found to answer a very good purpose.

The physiological effect of a full dose of hyoscine, say gr. 1-60, is manifested within twenty minutes. These are—brief, transitory bewilderment; marked interference with co-ordination; widely dilated pupils; slow, regular, very full pulse; dryness of the throat; relaxation of the vocal cords; very slow, full respirations, sometimes becoming Cheyne Stokes; marked suffusion of the face and of the general surface of the body; slight rise in temperature; and free diaphoresis, which does not seem to restore normal temperature. There is general muscular relaxation and a sense of wretchedness. Sleep usually follows, which continues from one to five hours, if the dose is given in daytime and the patient is put to bed. This amount, administered at night, would be followed by sleep lasting from eight to ten hours. The mydriatic effect is rather transient, but usually persists through an entire day. The pulse is slowed about twenty beats per minute, and this effect gradually wears away during eight or ten hours, and is often followed by a very variable period of pulse-acceleration, which seems to be simply a reactionary hastening of the pulse to restore the circulation. The normal rate of respiration is gradually restored through a period ranging from three to five hours.

The rise in temperature is not an invariable result, and is frequently small,—seldom exceeding one and a half degrees,—and the balance of temperature is usually restored within two or three hours. Dryness of the throat often persists through an entire day. Suffusion of the surface of the body is usually transitory. In moderate and in small doses the effect is of course, proportionate to the amount employed; but the same general symptoms are present, and the patient is quieted accordingly. Interference with the appetite is sometimes observed. It does not seem to act upon the bowels nor upon the kidneys.

Hyoscine is not always well borne; occasionally the following symptoms have followed the use of a moderate dose: nausea, vomiting, anorexia, dysuria, syncope, with small, rapid, irregular pulse, and symptoms of partial paralysis of the pneumogastrics.

Is it best to give the remedy hypodermically or by the mouth? It acts almost immediately and in rather less dose by the former method; yet, Dr. Wetherill prefers usually to give it by the mouth, as it acts very promptly, when taken into the stomach, even in very small doses, and this methcd offers no shock to an excited or timorous patient.

**The Galvano-Cautery as an Effectual Treatment of Diphtheria.**—(Therapeutic Gazette) That all therapeutic interferences at present employed in diphtheria play little more than a palliative role, without in any way modifying the diphtheric process itself, is unfortunately an indisputable fact with all clinicians and practitioners. To look for new remedies of the vegetable or mineral kingdom, or the products of the chemical laboratory, which might possibly exert a specific, or at least a salutary, influence over the affection, must at last appear a fruitless endeavor. Drugs do not and cannot cure diphtheria, and other measures must be thought of, unless we wish to abandon our patients wholly to the mercy of this dreaded foe. Almost simulteneously, and certainly independently of each other, the report of the successful exhibition of a new remedial procedure, viz., the galvano-cautery, comes to us from two different quarters. Dr. Bloebaum presented his experience with the galvano-cautery in diphtheria in a recent number of the Deutsche Medizinal Zeitung (p. 973, 1885), and expresses his unqualified satisfaction with the results obtained, while in the Rivista Venet of November, 1885, we find a similar eulogy of this treatment by Dr. Tedeschi (Communicazione Preventiva del Dott. v. Tedeschi di Trieste).

These authors declare that the application of the galvano-cautery does not produce the slightest pain, as the diphtheritic membrane is of course void of sensibility. At the mere touch of the glowing wire the membrane rolls up and falls off. Tedeschi emphasizes the fact that the once cauterized portion never again assumes a diphtheritic nature, and the application forms at the same time a positive check to the extension of the process over the neighboring parts. After the application of the cautery the fever is found to be sinking, and frequently to wholly disappear after two to four hours. At the same time the glandular swelling on the neck and the œdema of this region are decreasing.

The scab resulting from the cauterization falls off in eight to fourteen days, and always with ample suppuration. In the first couple of hours after the application ice is found to be a useful means to limit the inflammation; later, injections of aqueous vapor are indicated to favor a rapid dissolution of the scab. Irrigations with lime-water proved a useful adjuvant to this treatment.

Unfortunately, our author has not stated how many cases have been treated by this application of the cautery. But in the face of our present therapeutic impotency in diphtheria, the method deserves earnest but cautious trial. In conclusion, we recall that the galvano-cautery is not an entirely new application in the treatment of diphtheria, but has as early as in 1857 been recommended by French military surgeons in a communication to the Union Medicale.

**Fractures.**—Dr. Monette (Journal American Medical Association) says: As regards the fracture of the femur in children, suppose we have a case in an infant six or eighteen months old. What can be more cruel than a straight splint? My plan for treating these cases is to put them in a flexed position, similar to the sitting posture, and with extension of the anterior and posterior splints about the waist, and held by a band, and the lower extremities of the splints carried to or beyond the toes. By this method there is the most rigid immobility of the limb, and with comfort to all parts enveloped by the bandages. My first application of this form of splint was in the spring of 1868, and whenever a suitable case presents I utilize the identical splints, anterior and posterior, in conformity to a sitting posture.

Each day develops some ingenious contrivance for every form of fracture, but it is incumbent upon the surgeon to dispense with inventions to a great extent and utilize his ingenuity conformably with indications of his particular case; and there will be neither fault nor deformity.

Fractures of the clavicle offer some discouaging results, as the movements of the thorax (as in lying down) are prone to militate against permanent adjustment of any sort of dressing. It seems to me that a little more simplicity is requisite; for instance, simply binding the arm to the thorax by a bandage. In fractures of the clavicle in infants and very young children, the former particularly, I have pinned the sleeve of the arm of the fractured side to the dress anteriorly, or about the median line across the chest. Such treatment has resulted successfully, and with no more prominence of a callus than from the routine Fox apparatus, or that of any other specialist in surgical appliances. This confinement is readily tolerated by children, and a little pain causes them to bear it patiently. The nature of the fracture in children tends to confirm my method. With diminished ossific development, the fracture is not distinct; hence the reapposition of splintered fragments is readily accomplished; solidification takes place promptly, and immobility is attained sooner than in similar fractures in adults.

I need not mention other fractures, as simplicity can be more readily appreciated in those enumerated than in those not mentioned, as the form of treatment is absolutely indicated. Recovery and restitution are much more speedy and satisfactory, and at the same time careful inspection can be made by the attendant at opportune periods, without fear of compromising the progress of bony union.

**The Thermo-Cautery with Treatment of Spontaneous Gangrene.**—(St. Louis Med. and Surg. Journal) M. Verneuil (Le Progress Medical) maintains that surgery possesses in thermo-cautery a remedy which leaves far behind the bistoury with its disastrous results in cases of idiopathic gangrene. He relates a case of a man of 54 years of age, who, after paring a corn on his foot saw spontaneous gangrene set in. An attempt was made to circumscribe it with the thermo-cautery, but new gangrenous plaques made their appearance. The discovery was made that the patient was glycosuric and an alcoholist. M. Verneuil remarked that gangrene never advenes in the diabetic, unless they are given to alcholism. Choparts amputation was made, with rapid recovery, and gangrene never recurred. The conclusion reached is, that spontaneous gangrene should be treated with the thermo-cautery, to the exclusion of the bistoury.

**Passage of a Large Piece of Glass Through the Foot, After Five Months.**—Dr. Allaben, in Journal American Medical Association:

William L. D., aged 17 years, while swimming on July 2, 1885, cut a deep gash in his right foot on some sharp object in the water. The wound was about the center of the plantar surface of the foot, from side to side, and about an inch and a half from the base of the toes. There was considerable hæmorrhage, which was arrested by a companion. The wound was completely healed in two weeks, and no physician had seen the case. About this time a small swelling appeared on the top of the foot, causing great sensitiveness, on account of which the top of the shoe had to be cut away. This was the only inconvenience that the patient suffered, except that when he had been on his foot all day it became tired and somewhat painful. He could walk and run without difficulty or limping, and for five months the right foot was used as much as the other.

On November 12, the sensitive spot on the top of the foot was accidentally struck, causing great pain and rupture of the skin, giving exit to some blood and pus. I was called in on the evening of November 13. Near the surface of the small open wound I found the point a hard foreign subtance, which on probing was found to be of considerable size, and deeply imbedded in the foot. Attempts at removal caused such pain that it was necessary to administer chloroform. The opening, which corresponded with the space between the second and third metatarsal bones, was enlarged, and with a pair of dressing forceps considerable force was required to dislodge the foreign body, which proved to be a triangular piece of glass, measuring three-quarters of an inch on every side, and one-eighth of an inch in thickness. It is evident that this piece of glass had been in the foot for more than five months, and had passed completely through the member. The boy is now recovering rapidly.

There are two things that seem quite remarkable to me in this case: 1. That a foreign body of this character could pass entirely through the foot between the metatarsal bones without causing more interference to locomotion. 2. That such a substance could remain in an organ so much needed as the foot, without exciting more inflammation and suppuration.

**Hypodermic Injections of Cold Water in Sciatica.**—(Therapeutic Gazette) Dr. H. D. Lewis, of Lone Pine, Pa., writes to the New York Medical Record for January 23, 1886, that he was consulted by a man of 60 years of age, who was suffering greatly from sciatica. He had been treated for the past eight weeks by two physicians, and had run through the entire list of anti-neuralgic remedies. Being desirous of trying something which was at least new to the patient, Dr. Lewis determined to employ hypodermic medication, and having no drug handy which he cared to use, he filled the syringe with cold water and injected the fluid deep down behind the trochanter. The following day the patient returned and said that he was feeling much better. The injections were accordingly repeated every third or fourth day for a period of three weeks, by the end of which time a complete cure was obtained. The writer has since treated a number of cases of sciatica in the same way, with equally gratifying results. He thinks that possibly many of those cases which have been reported as cured by the injection of certain drugs, such as cocaine, might have terminated in an equally favorable manner had simply cold water been used.

**Hypnone—A New Hypnotic.**—(St. Louis Medical Journal) M. Dujardin Beaumetz announced to the Academie de Medicine at a recent seance, the fact that *methylphenylacetone*, when taken in doses of from four to ten minims, has very powerful hypnotic properties—a fact which dictated the shortening of its chemical name into the more suggestive *hypnone*. Curiously enough, fractional doses have no such effects, or display them but slightly. Two minims of the fluid weigh five centigrams or about three-quarters of a grain, hence the dose should be from a grain and a half to four grains. It may be administered in capsules, or as suggested by M. Vigier, the veteran pharmacist to whom the physicians of the world owe so much for his labors in the pharmacy of new remedies. In the Gazetee Hebdomedaire for Nov. 27, 1885, he offers the following formulæ for a syrup and an elixir of hypnone.

*Syrup of Hypnone:* For each minim of the remedy take 15 minims of alcohol of 90°. 75 minims of syrup of orange flowers and 15 minims of syrup of cherry laurel; mix the hypnone with the alcohol and add the syrups. This will keep any length of time. For extemporaneous prescriptions it would seem to me that much less syrup would be advisable.

*Elixir of Hypnone* is made by dropping the hypnone into alcohol of 60° (45 minims of alcohol to each minim of hypnone) and adding an equal quantity of syrup of mint.

[Hypnone, or methyplhenlacetone, although so recently introduced into medicine, is not a new chemical, by any means. It was discovered by accident, as long ago as 1857, by Prof. Friedel (a Frenchman, in spite of his Teutonic name) while distilling a mixture of acetate benzoate of calcium. At ordinary temperatures it is a liquid somewhat denser than water (s. g. 1,032), having a pungent taste and an odor which reminds one about equally of oil of wintergreen and oil of bitter almonds. At 60° it crystalizes in large tabular crystals; it boils at about 390° F. and has the formula $C_6H_5(CO)(CH_3)$.—F. L. J.

**Keloid.**—(Maryland Medical Journal) In the Surgical Society of Paris, Dr. Reclus cited a case in which he had obtained great amelioration by local compression with mercurial plaster (*emplatre de Vigo,*) salt baths, and cod-liver oil in large doses internally. Dr. Ledentu and Dr. Berger also spoke of *crossed* linear scarifications carried out according to the method of Dr. E. Vidal. So far as I am concerned, I am convinced that this last procedure is by far the best, or if you prefer the least objectionable, only you must know how to apply it. Each week parallel linear incisions must be made through the whole thickness of the tumor, and passing beyond its borders, and these must be crossed at right angles by other similar incisions, in such a manner as to form little squares. According to my idea, these incisions should be as close together as possible between the operations, *emplastrum de Vigo cum mercurio* should be kept constantly applied. I have already treated three keloids after this manner. In the first case, the tumor was very much reduced in size, but did not entirely disappear despite a great number of operations. In the second case, where we had to do with two keloid tumors of the right cheek, consecutive to caustic applications, eight scarifications have been sufficient to cause their disappearance, but I am keeping the patient under observation, expecting a reoccurrence of the disease.

The third case is one of enormous keloid, having a diameter of ten centimetres in all directions, and a thickness of one and a half centimetres, situated a little above the pit of the epigastrium. Here I have already done more than forty scarifications, obtaining a diminution of at least five-sixths of its thickness, but I am far from having produced a complete disappearance of the growth. One of the great advantages upon which Dr. Vidal has insisted, is that of rendering the keloid indolent when it is causing suffering to the patient, which it not infrequently does.

**Abortion from Sassafras.**—Dr. John Bartlett read a paper, entitled Remarks on the toxic properties of Sassafras, before the Chicago Medical Society. He stated that, for some years past, he had an intention of bringing before the profession reasons, rather feeble it must be admitted, for the supposition that the medicine under consideration has marked potency in a direction, so far as he knows, not suspected by medicine men. Up to this time the declaration on the part of standard writers that sassafras is a remedy of questionable power, and the fact that it is hawked about the streets and used freely as a tea all over the country, has caused him to refrain from bringing before a scientific body his limited experience presently to be detailed. But the recent declaration that this drug possesses toxic properties may justify him in making the following statement:

Years ago, he was called to a woman among the poorer classes, of good intelligence and education, who was having a miscarriage. Upon inquiring as to the cause of the mishap, with a prefatory reference to her poverty and already large family, she stated that she had induced the abortion herself—that she had done so on previous occasions. She had employed, she said, "what other women used, sassafras tea." She was surprised that he did not know of the property of sassafras as an oxotoxic. She spoke as if all her friends knew how to use it as an ecbolic, and she evidently looked upon it as a specific. Tea, she said, made from four or five pieces of the root, as large as the thumb and twice as long, would produce abortive effect.

[Other cases are cited in the paper of Dr. Bartlett, as indicating that sassafras has an abortive property. We mention it as an item which the profession may test, but have no faith in it.—EDITOR SOUTHERN MEDICAL RECORD.]

**Removal of Blade of Tooth-Forceps from the Right Bronchus.**—(New Orleans Medical and Surgical Journal) The following case was reported to the Odontological Society of Great Britain, by Sir William MacCormac, and published in the British Medical Journal:

In an attempt to remove the right upper second bicupsid, the palatine blade snapped off close to the joint and disappeared. "The patient was at once seized with difficulty of breathing and lividity of the face, and it appeared for a time as if she would die of suffocation. It being evident that the fragment had entered the larynx, prompt measures were taken to favor its expulsion; the patient was inverted, and an emetic given, but without success. The alarming symptoms gradually passed off, and during the next five or six weeks, the patient suffered only from paroxysmal cough, pain on the right of the sternum opposite the second intercostal space, and dyspnœa on exertion. These symptoms becoming more marked, with bloody expectoration and evident loss of strength, the patient was at last, seven weeks after the accident, sent to London, and admitted into St. Thomas' Hospital. The ausculatory signs pointed to the presence of the foreign body in the right bronchus. Chloroform having been administered, Sir William MacCormac exposed the trachea from the cricoid cartilage almost to the sternal notch, divided the isthmus of the thyroid body, and made an opening an inch and a half long into the windpipe. On passing down a probe, the steel fragment could be distinctly felt in the right bronchus, at a distance of about four inches from the lower end of the wound, and about an inch and a half beyond the bifurcation of the trachea. A polypus-forceps was then introduced, and after a great deal of difficulty, caused by the wedge-like shape and smooth surface of the fragment, it was at length seized and extracted. It measured an inch in length. The patient had some slight broncho-pneumonia, but made an excellent recovery.

**Opium Habit.**—A writer in the American Medical Journal says: The course we advise and adopt will prove incomparably superior to any substitution practice, and then when our patients are cured they are not left in subjection to any drug. What benefit is there in changing one habit for one scarcely less objectionable and sometimes even more disastrous in results?

In our treatment of the morphine or opium habit, if we gradually lessen the dose daily, giving barely enough to keep the patient in a tolerable condition, and see that he gets good nourishing food, and give him very small doses of chloride of gold and sodium—the one hundredth of a grain every three hours—with phosphate of potassium in alternation, we need not resort to substitutes, and yet we soon witness signs of improvement. As we withdraw the morphine and feed our patients, the renewal of life proceeds, and within a week or ten days—in one month at farthest—we can so completely renew them as to enable them to live without morphine. They may feel weak for a time, for they are comparatively new, and may suffer from temptations, but if due caution is observed they will enjoy a permanent cure.

The whole secret of curing the morphine habit is to know that the patient is partially dead—killed with morphine—and that he is not capable of curing himself; that you must restrain him and gradually withdraw the murderer, and permit a renewal of life to take place.

**Antipyrine in Epistaxis.**—(Medical Age) We have before alluded to the hæmostatic properties of anti pyrine. Dr. Lavrand, in the Journal de Medicine et de Chirurgie Pratiques, confirms other observers. It is certain and prompt in its effects, he says, and is much superior to perchloride of iron, as it is colorless and does not coagulate the tissues like the latter substance. It is used in aqueous solution, of the strength of 1 to 30, applied on lint and inserted as far as possible into the nares. The nostril is then compressed with the fingers so that the tampon soaked in the hæmostatic is in contact with a large extent of the mucous surface. By means of several applications thus made, M. Lavrand succeeded in arresting epistaxis which had persisted in spite of plugging of the anterior and posterior nares.

**Small-Pox.**—(Medical Bulletin) Dr. J. M. Jackson, (Mississippi Valley Medical Monthly) says: When called to a case of small-pox I always proceed to vaccinate at once and at the same time clear out the alimentary canal with saline cathatics as early as practicable. To break down congestion by equalizing circulation, and at the same time to counteract the much dreaded septicæmia, I administer quinine in 5 grain doses, combined with carbolic acid in half grain doses, to be repeated every four or five hours. Quinine is not only our most effective remedy against congestion, but is one of our best antiseptics.

As soon as the pustules are developed I take a hypodermic syringe and aspirate the lymph or pus, as the case may be, from time to time from the pustules. By this means I prevent the absorption of the greater portion of the pus, and thereby prevent, in a great measure, the formidable and fatal effects of blood-poisoning, in consequence of which we find the alarming symptoms of depression of the vital powers and secondary fever.

The question comes up, can the duration of small-pox be cut short by this? I answer, Yes, and materially so. In my practice I am satisfied the duration of the disease has been shortened five or six days, and even during its continuance all the most alarming symptoms have been greatly ameliorated. I at the same time apply a mixture of olive oil and carbolic acid, consisting of ten parts of the former to one of the latter, over the whole pustulated surface. This can be done with a soft swab, or, perhaps, better with a large feather.

**Peppermint vs. Cocaine.**—(Medical World) At a recent meeting of the Mexican Academy of Medicine, Dr. Miguel Cordero reported a number of cases in which he used peppermint as a local anæsthetic, showing it to possess remarkable powers. A case of intercostal neuralgia which resisted the usual means of relief, yielded completely and permanently to a hypodermic injection of four drops at the seat of pain. Another case similar to this, which had for a number of weeks resisted the usual treatment, including morphine hypodermically, yielded promptly and permanently to a hypodermic injection of four drops of the peppermint. A number of cases of facial neuralgia were cured by injection along the course of the nerves in the parotid region, or in the temporal region, if the neuralgia be ocular.

None of these injections were followed by inflamation or abscess.

In the discussion which followed the reading of this paper, the question was raised that the author had not told his formula for preparing the remedy for injection. This, strange to say, the author persistently refused to do, saying that as it was an old remedy each member should study up a mode of preparing it.

**Conium as a Nerve Stimulus.**—The Homœopaths laud the wonderful effect of small doses of conium in rousing into increased action the nerve force in old age, increasing the powers of digestion and checking the wasting of tissues.

**Amblyopia Nicotiana.**—(Medical Zietung.) De B. remarks on whether *amblyopia ex abuso* is caused by alcohol or tobacco. He found :

1. With three—none alcoholic—smoking women characteristic symptoms, diminished central vision, central color-scotoma with normal vision.
2. In woman, amblyopia from tobacco is rarer than from alcohol.
3. Smoking on an empty stomach, in enfeebled condition from whatever cause, promotes the development of amblyopia. Dyspepsia and insomnia may both be the cause and effect of smoking.
4. Good food and a moderate use of alcohol, antagonize the effects of tobacco.
5. Amblyopia nicotiana is a functional vaso-motor disturbance and not the product of a neuritis.—*St. Louis Medical Journal.*

**The Best Vehicle for Quinine Administered to Children.**—Dr. Kenner (New England Medical Monthly) says : "Until the syrup of yerba santa was put upon the market I had great trouble in finding a suitable vehicle for the exhibition of quinine to children. While the licorice, and the ulmus preparations, and the tannic acid formula were measurably useful, they were inadequate when large doses were given or when the quinine was to be used for a considerable time. I depend upon its use per orem till the stomâch had become intolerant to a solution of quinine in water and sulphuric acid, and then resorted to its use per enema. My experience with the syrup of yerba santa is such as to warrant me in saying that it deserves the confidence of the profession as a menstruum whereby the sulphate of quinine cannot only be rendered tasteless but absolutely palatable. Five grains of quinine is rendered tasteless in one drachm of the syrup.

**Ice to the Spine in Obstinate Vomiting.**—(Therapeutic Gazette) Dr. W. L. Davis reports (Mississippi Valley Medical Monthly) a case of vomiting in typhoid fever in which every remedy, even pellets of ice, was rejected by the stomach. He applied ice to the lower part of the spine in considerable quantity, and the vomiting instantly ceased ; a profuse perspiration followed. The use of ice was only persisted in when indicated ; and cool sponging was instituted with marked benefit, so that the ice was only occasionally required. Recovery in the average time took place.

**Determination of the Sex of the Fœtus.**—(Indiana Medical Journal) Senor Juan B. Bidart has examined one hundred women, toward the termination of pregnancy, with a view of determining the sex of the child before birth. He concludes that when the number of beats of the child's heart is below 135 a minute, the child is usually a boy, and when the number is above 145 it is generally a girl. He diagnosed the sex correctly in 92% of the cases. When the beats were between 135 and 145 it was quite impossible to do more than guess at the sex.

**Abortive Treatment of Gonorrhœa.**—In the Monatsschrift fuer Pratische Dermatologie, the results of abortive treatment, by means of resorcin, in 180 cases is given. A. J. Munnich, the reporter, says that he employs in fresh cases a 3 per cent. solution of resorcin, and orders injections of such a solution to be made every two hours during the day and at least twice at night, a syringeful each time. The patients are directed to drink freely of milk and water, and urine is to be voided, if possible, before each in-injection. After four or five days the discharge is usually much diminished, and then three or four injections by day and one by night suffice. Munnich claims sixty-seven complete successes of the whole number so treated. The average duration of the treatment was two weeks. The resorcin should be chemically pure and the solution kept in a dark vial.—*Exchange.*

**Treatment of Otorrhœa.**—(Berlin Klin. Wochenschr.) Bruckner recommends two mercurial preparations for combating otorrhœa, (not dispensing with boracic acid, arg-nitr, alcohol) but to add to them. The red precipitate is applicable only in large perforations of the membrani tympani and only after scrupulous—not dry—cleansing. But the sublimate is of great value in copious feted blenorrhœa ; it is best used by instillation in an alcoholic solution (0.05—0.1: 50.0) We must be grateful for every new remedy in otorrhœa. Justly does boracic acid occupy the first rank in the therapeutics of suppurative inflammation of the ear. Yet there are many cases known where its effects were only negative. Then the sublimate may be resorted to, with greater probability of a good result.—*St. Louis Medical Journal.*

**A New Electric Plant.**—Phytolacca electrica (says the Medical Times) is the name given to a plant which possesses strongly marked electro-magnetic properties. In breaking a twig the hand receives a shock that resembles the sensation produced by an inducting coil. Experiments made on this plant showed that a small compass was affected by it at a distance of about twenty feet. On a near approach the needle vibrated, and finally began to revolve quite rapidly. The phenomenon was repeated in reverse order on receding from the plant. It is said that no birds or insects are ever seen on or about this plant. The soil where it grew contained no magnetic metal like iron, cobalt, or nickel, and it is evident the plant itself possesses this electrical property.

**Morphine in Infantile Eclampsia.**—(St. Louis Medical and Surgical Journal) Dr. Levantiner, of Constantinople, details in the Centralblatt fuer klinische Medicine a case of infantile convulsions which had resisted hot baths, cold compresses, chloral, chloroform, etc., and had continued with increasing frequency and strength for many hours, but which yieded promptly and completely to hypodermic injections of morphine. The child was four months old and the amount of morphine injected at one time was about one-twentieth of a grain. Four injections in all were given, no other medication being resorted to.

**Hot Cloths for Uterine Hemorrhage.**—Dr. G. Henri Bogart, of Sharptown, Ind. (in the Medical Age), says of a case of post partum hemorrhage: I first tried the hot cloths over the uterus in a lady, delivered by an old midwife. Moderate post partum hemorrhage had been treated with cinnamon, quinine, hot coffee, cold applications, and kneading of uterus; and, when I arrived, I put a little ergot on top of the previous multiple treatment, but unsuccessfully. Hot cloths produced good contractions in ten minutes, with cessation of flooding.

**Glycerin in Colds.**—(National Druggist) When suffering from a "cold" with tumefied, hot, and burning nostrils, and pain over the eyes in the frontal sinuses, much relief is often obtained by introducing glycerin into the nostrils with a camel hair pencil. The glycerine will spread over the entire mucous membrane of the nose, which then becomes moist, and the tumefaction, which may have completely occluded the nostrils, subsides, and the respiration through the nose again becomes possible, while the pain is much ameliorated or entirely disappears.

**Restoring Gray Hair to its Original Color.**—We have under our notice (says the Medical World) a patient whose prematurely gray hair is slowly but surely returning to its original color, under the internal administration of phosphorized cod-liver oil (which is being taken for another purpose). We have observed this effect of the phosphorus and oil before now, and are inclined to think that the hopes of success are more rational from this treatment than from the use of any of the so-called hair restorers.

**For Hay-Fever and Coryza.**—The London Lancet recommends (says the Popular Science News) the following prescription in the form of powder, a small quantity to be puffed into the nostrils by means of an insufflator, which can be procured of any instrument maker, or by one of Dr. Horace Bobell's tubes: Acid boracici pulv. ʒ ss.; sodæ salicylatis, ℈ ij; cocaini hydro-chlor, gr. ij. He has also found that this prevents coryza if used at the onset of an attack; namely, the sneezing stage.

**Coryza.**—(Medical Age) It is said that this troublesome affection may be promptly relieved by the introduction into the nasal cavity, six times daily, of two drops of a two per cent. solution of the hydrochlorate of cocaine. Children which previously have obstinately refused to nurse will commence to suckle a few minutes after the first application of the cocaine, and the coryza is ordinarily cured after about four days of this treatment

**The Action of Quinine on the Fœtus.**—Vadenuke, in a These inaug. de St. Petersburg (New York Medical Journal), arrives at the following conclusions: 1. Quinine taken by the mother passes into the system of the fœtus in the proportion of about one-ninth of the whole amount. 2. The largest amount is contained in the fœtus at the end of two hours. 3. The fœtus eliminates it in a little more than forty-eight hours, and the neonatus in seventy-two hours. 4. Large therapeutic doses given to the mother do no harm to the fœtus. 5. The same is true of large doses repeated every forty-eight hours. 6. Quinine is not an abortifacent. 7. It may ward off abortion or premature labor when the mother is under the influence of fever, especially of malarial origin.

**External Use of Lobelia Inflata.**—Dr. V. N. Reichard highly recommends the use of lobelia inflata (Medical Times, December 12, 1885) as a local application for indolent sores, chronic erysipelas, and especially in incise wounds, in which latter class of cases it acts as a hæmostatic and astringent. The mode of employment of lobelia for this purpose in cases of incised wounds, no matter how great the hemorrhage, provided, however, it does not require a ligature, is to bring the edges of the wound together, and to hold them for a few moments, while a pledget of cotton, wet with tincture of lobelia, is applied. Dr. Reichard says that the hemorrhage will then cease and the parts adhere, and although lobelia may not be a germicide, it will so entirely close up a wound as to render it perfectly aseptic.

**Exenteration of the Eye.**—(New Orleans Medical and Surgical Journal) Dr. B. E. Fryer, of Kansas City, Mo., in a paper (Kansas City Medical Record, December), discussing this operation expresses himself as being inclined to doubt its superiority to enucleation. He mentions besides Knapp's case (see this Journal, October number, p. 308), a case in which enucleation had to be performed on account of sympathetic irritation, although *abscision* of the anterior portion of the globe had been done eighteen years ago. It is not stated, however, whether the choroid, retina, etc., were removed at the time the abscision was done. This is *the* important point, for such removal of the contents of the globe constitutes the essence of exenteration.

**Borated Vaseline in Erysipelas.**—Dr. Romit (Revista Clinica, 1885,) speaks very highly of boracic acid in vaseline, 1 part to 20, as an application to the inflamed skin in traumatic erysipelas. It has an advantage over certain other applications, in that it causes no discoloration, and does not mask the redness of the skin, so that the progress of the disease toward recovery can be readily observed. The redness and tumefaction rapidly disappear, the pain is quited, and a cure is obtained within three or four days. The application is made over the affected part, and for some distance around over the healthy integument, and is repeated morning and evening.

DIPHTHERIA is constantly increasing in England. In 1880 the number of deaths registered as due to this disease amounted to 2,612; in 1881, they were 2,976; 1882 exhibited a further increase of 3,756; in 1883 they reached 3,976; and during 1884, the last completed year, they were no less than 4,696.

